# Association between cardiometabolic index change patterns and incident cardiovascular disease in middle-aged and older adults

**DOI:** 10.3389/fcvm.2026.1845385

**Published:** 2026-07-31

**Authors:** Hongyan Li, Hongliang Tian, Haitao Dong, Hui Gao, Bo Yang, Shijie Kou, Hongmei Tian

**Affiliations:** 1 Department of Cardiovascular Medicine, Affiliated Hospital of Chifeng University, Chifeng, China; 2 Department of Cardiology, Zibo Central Hospital, Zibo, China; 3 Graduate School, Dalian Medical University, Dalian, China; 4 Department of Cardiovascular Medicine, The First People’s Hospital of Shangqiu, Shangqiu, China

**Keywords:** cardiometabolic index, cardiovascular disease, change pattern, cumulative effect, trajectory

## Abstract

**Purpose:**

No evidence exists regarding the association of long-term cardiometabolic index (CMI) changes with cardiovascular disease (CVD). Our aim is to investigate the association and dose-response relationship between cumulative and long-term changes of CMI and incident CVD risk.

**Materials and methods:**

The study participants were drawn from the China Health and Retirement Longitudinal Survey (CHARLS) and English Longitudinal Study of Ageing (ELSA) cohorts, and Electronic Medical Records (EMR) in a hospital of Inner Mongolia, China. K-means clustering was used to identify CMI change patterns. Cox proportional hazards models were employed to assess the association between cumulative CMI and CMI change patterns and incident CVD risk. Restricted cubic spline (RCS) analysis was used to account for the nonlinear relationship of cumulative CMI.

**Results:**

Univariate Cox models revealed a significantly higher CVD incidence risk in the higher CMI trajectory groups. This association remained significant after adjusted. Compared with the lowest CMI group, the hazard ratios (HR) for incident CVD in the highest CMI group were 1.82 (95% CI: 1.47–2.26), 1.92 (95% CI: 1.30–2.85), and 2.00 (95% CI: 1.45–2.76) in the three cohorts, respectively. Cumulative CMI was associated with increased CVD risk, with HR of 1.05 (95% CI: 1.01–1.09), 1.15 (95% CI: 1.07–1.23), and 1.04 (95% CI: 1.01–1.07), respectively, and a nonlinear dose-response relationship was observed.

**Conclusion:**

Persistently elevated CMI levels are significantly associated with an increased risk of incident CVD among middle-aged and older adults. Long-term monitoring and management of CMI contribute to reducing CVD incidence and improving prognosis.

## Introduction

Cardiovascular disease (CVD) remains the leading cause of premature death and disease burden globally (1, 2). According to the Global Burden of Disease (GBD) Study, the prevalence of CVD and the number of deaths increased considerably from 1990 to 2019 (3, 4). CVD includes a range of circulatory diseases such as ischemic heart disease, stroke, coronary heart disease, and heart attack, which are associated with an increased risk of non-cardiovascular and all-cause mortality (5). The burden of CVD increases progressively from the age of 30 years and peaks around the age of 65 years (3). Due to the complexity of CVD etiology, regional disparities persist, despite CVD becoming a major global concern. Therefore, early identification and intervention of risk factors play a key role in controlling the CVD epidemic.

Traditionally, various anthropometric and metabolic indicators have largely been used for CVD risk stratification. Anthropometric measures such as body mass index (BMI) and waist-to-height ratio (WHtR) primarily reflect systemic or abdominal fat accumulation but cannot simultaneously reflect underlying lipid metabolism disorders or insulin resistance (IR) (6, 7). Conversely, biochemical surrogate markers such as the triglyceride-glucose (TyG) index and lipid accumulation product (LAP) have gradually emerged as reliable indicators for assessing insulin resistance and arterial stiffness (8, 9). However, these markers often overlook the synergistic relationship between visceral obesity and systemic dyslipidaemia. The cardiometabolic index (CMI) is a recently proposed comprehensive metabolism-related index of visceral obesity that is strongly associated with obesity-related metabolic disorders (10). The CMI is calculated as the product of the triglycerides to high density lipoprotein cholesterol (TG/HDL-C) ratio and the WHtR (11). Both are important components of the metabolic syndrome (12). CMI has been suggested as a valuable indicator for identifying metabolically obese adults with normal body weight (13). The CMI offers distinct clinical advantages by combining the WHtR with the triglyceride-to-high-density lipoprotein cholesterol ratio. Nevertheless, unlike the TyG index, the CMI does not take glucose metabolism parameters into account. Several epidemiological studies have found significant associations between CMI and various diseases, including acute pancreatitis (10), depression (14, 15), cardiometabolic multimorbidity (16), diabetes (17), congestive heart failure (18), and stroke (19). Cohort studies based on the National Health and Nutrition Examination Survey (NHANES) and the Sleep Heart Health Study (SHHS) have provided evidence that CMI is associated with increased CVD mortality (20, 21). However, prospective epidemiological evidence establishing the longitudinal association between CMI and incident CVD remains limited. Previous research has been largely confined to cross-sectional study designs or has focused on specific high-risk clinical subgroups, such as patients already suffering from hypertension, type 2 diabetes or peripheral arterial disease (22–24). There remains a lack of studies between the CMI control levels and CVD. Evaluating the long-term dynamic changes in CMI control levels through prospective cohort studies can provide crucial insights into dynamic risk indicators for the CVD prevention.

Epidemiological studies have consistently shown that the incidence of CVD is strongly age-dependent, with individuals aged ≥ 45 years demonstrating higher susceptibility to the disease (3, 25). Therefore, early identification of modifiable CVD risk factors in the middle-aged and elderly populations is crucial for CVD prevention. Our study aimed to explore the association between CMI control levels, the combined effect of CMI and BMI and the risk of incident CVD in a middle-aged and elderly population through two large prospective studies.

## Materials and methods

### Study design and population

**Figure 1:**
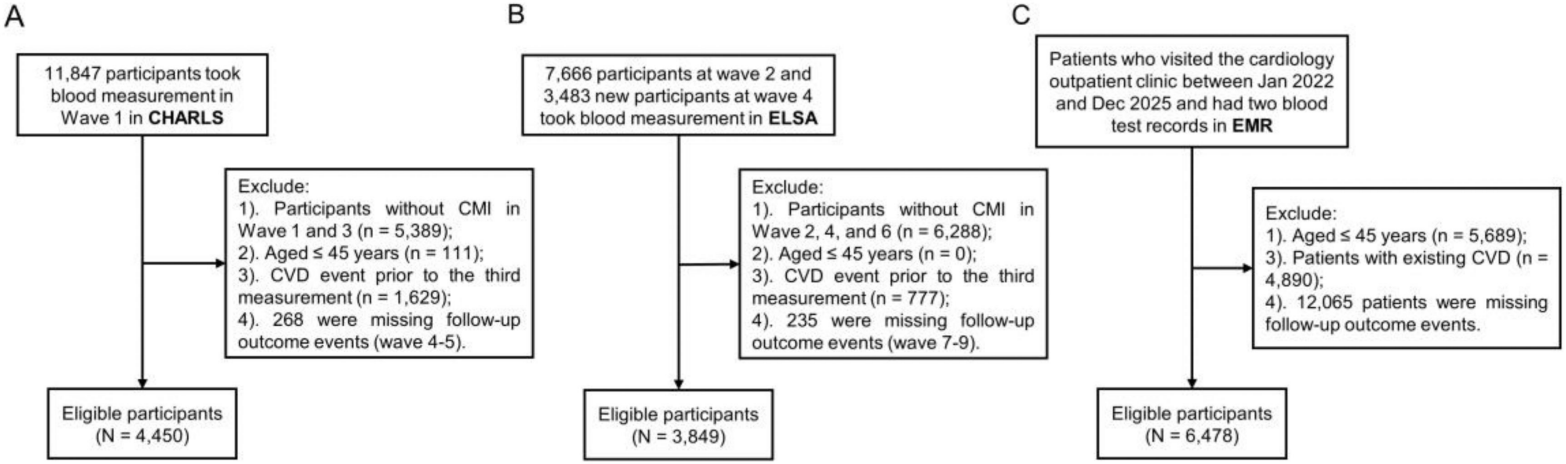
Process for screening participants included in this study from three datasets: CHARLS **(A)**, ELSA **(B)**, and EMR **(C)**.

Study data were drawn from the Chinese Longitudinal Study of Health and Retirement (CHARLS), the English Longitudinal Study of Ageing (ELSA), and Electronic Medical Records (EMR) from an inpatient department at a hospital in Inner Mongolia, China. CHARLS and ELSA are prospective cohort studies focusing on community-dwelling adults aged 45 and older in China and 50 and older in the United Kingdom, respectively. Details for the study designs and methods of the two cohorts have been described elsewhere (26, 27). In the CHARLS dataset, participants underwent blood biochemistry at wave 1 (2011) and wave 3 (2015), and disease were recorded at all five waves (2011, 2013, 2015, 2018, and 2020). We obtained records of blood tests at wave 2 (2004) and wave 4 (2008), or wave 4 and wave 6 (2010) to evaluate changes in CMI and cumulative CMI in the ELSA. The study timeline is presented in Supplementary Figure S1. Exclusion criteria comprised the following: (1) individuals without triglyceride, HDL-C, waist circumference, and height measurements at the first and second biomarker measurements; (2) aged ≤ 45 years; (3) CVD event prior to the third measurement; and (4) absence of a documented CVD event at the end of follow-up. Our EMR data were drawn from electronic records of hospital visits between January 2022 and December 2025. We screened medical records of patients aged 45 years or older who had at least two blood tests and at least one subsequent diagnostic record to identify patterns of CMI change and incident diseases. A detailed step-by-step flowchart illustrating the comprehensive participant selection, including specific exclusion numbers at each stage across the CHARLS, ELSA, and EMR cohorts, is systematically presented in Figure 1.

The CHARLS was approved by the Biomedical Ethics Committee of Peking University (IRB 00001052-11015). ELSA was approved by the London Multicenter Research Ethics Committee (MREC/01/2/91). The EMR dataset is a retrospective observational study utilising de-identified and anonymised data extracted from a standard hospital information system (HIS), requiring no patient participation. Consequently, the Ethics Committee of the Affiliated Hospital of Chifeng University has formally waived the requirements for formal ethical review and written informed consent. All data collection and analysis procedures for the three cohorts were conducted in strict accordance with the ethical principles of the Declaration of Helsinki and relevant national guidelines on confidentiality in biomedical research.

### Cardiometabolic index measurements

CMI was calculated using blood biochemicals and physical examination measurements, including, triglycerides (TG, mmol/L), high-density lipoprotein cholesterol (HDL-C, mmol/L), height (cm) and waist circumference (WC, cm). The formula for the calculation of CMI is as follows: $CMI=\frac{TG}{HDL-C}\times \frac{WC}{Height}$. The cumulative CMI was expressed as the mean of the two measurements multiplied by the interval scale: (CMI_1st_ + CMI_2nd_)/2 × time (Time_1st_ − Time_2nd_). As both the CHARLS and ELSA cohorts employed a structured, fixed-time prospective follow-up design, the assessment intervals for all participants included in each cohort were identical: 4.0 years for the CHARLS cohort and 2.0 years for the ELSA cohort. For the EMR cohort, the median interval between assessments was 20.5 (IQR: 13.5–22.0) months.

### Assessment of cardiovascular disease

The primary outcome in our study was CVD events, with heart disease and stroke as secondary outcomes. Self-reported or diagnosed CVD events were collected for participants at each wave of CHARLS and ELSA. In CHARLS, participants were interviewed by asking “Did your doctor tell you that you have been diagnosed with heart attack, angina, coronary heart disease, heart failure, or other heart problems?”, and “Did your doctor tell you that you were diagnosed with a stroke?” to identify CVD events. Similarly, in the ELSA cohort, incident CVD events were identified using standardized, structured interview questionnaires administered at each follow-up wave. Participants were explicitly asked whether a doctor had ever informed them that they had been diagnosed with any of the following specific conditions: heart attack (including myocardial infarction or coronary thrombosis), angina, congestive heart failure, or stroke. While community-based cohorts (CHARLS and ELSA) relied on validated self-reported physician diagnoses during longitudinal follow-up waves, clinical diagnoses of incident CVD in our EMR dataset were strictly confirmed and defined using International Classification of Diseases-9/10 (ICD-9/10) diagnostic codes.

### Covariates

Common features across three databases, along with previous studies, were used to identify potential confounders at baseline. Demographic variables included age (<60 and ≥60 years), sex, education level [(primary or lower, and secondary or higher in CHARLS and EMR) and (O-level or below, and higher than O-level in ELSA)], and marital status (Spousal/Others). Health behavioral factors included smoking status (Yes/No), recent alcohol consumption (Yes/No), body mass index (BMI, categorized as ≤25, 25–30, >30 Kg/m^2^), diabetes diagnosis (Yes/No), systolic blood pressure (SBP, mmHg), and diastolic blood pressure (DBP, mmHg). Blood biochemicals included total cholesterol (mmol/L), C-reactive protein (CRP, mg/L), Triglycerides (TG, mmol/L), Low-density lipoprotein cholesterol (LDL-C, mmol/L), and High-density lipoprotein cholesterol (HDL-C, mmol/L).

### Statistical analysis

**Figure 2:**
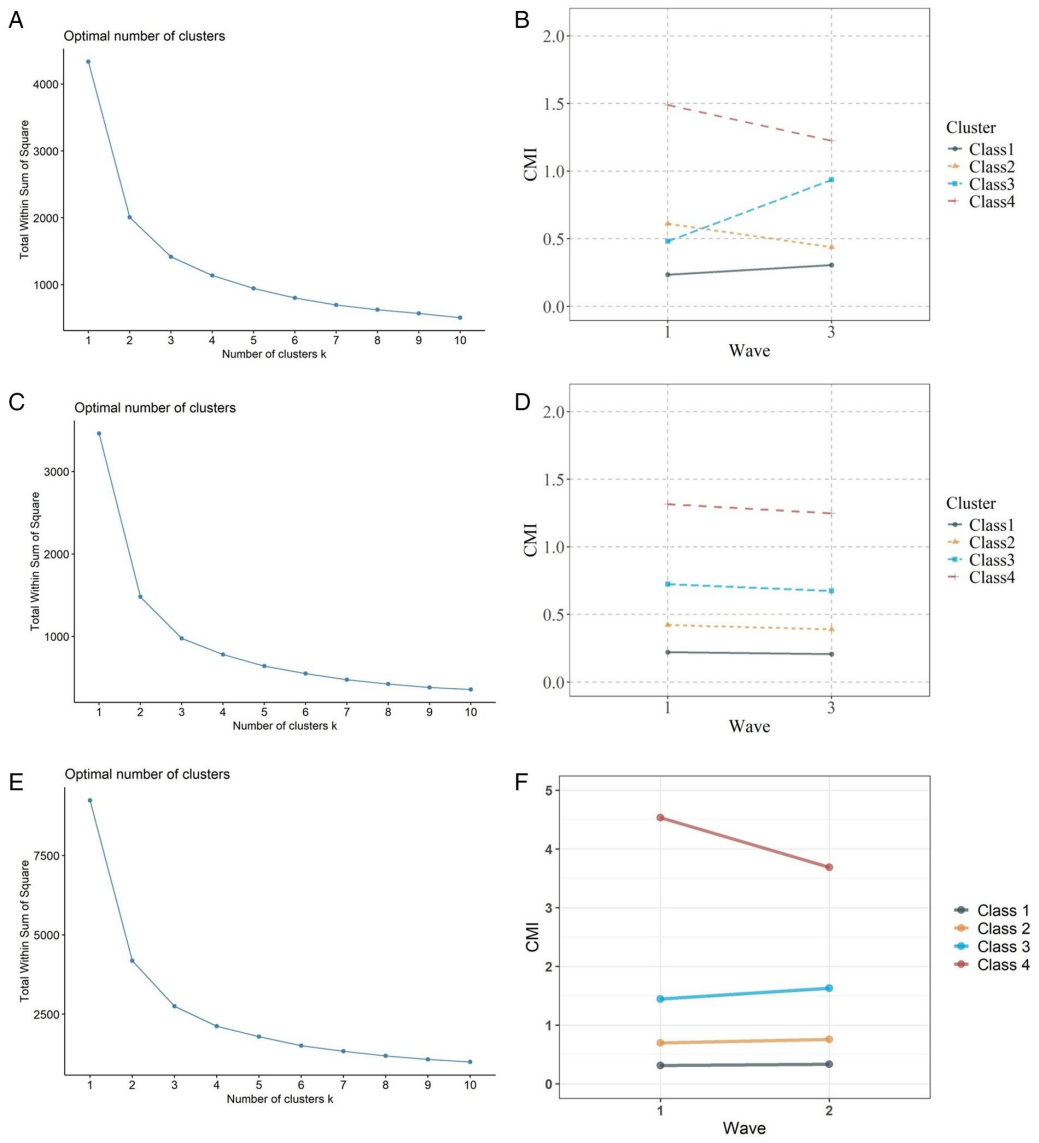
Clustering the cardiometabolic index of participants in the CHARLS **(A,B)**, ELSA **(C,D)** cohorts, and EMR **(E,F)** datasets using the K-means method.

As our longitudinal dataset assessed biomarkers twice for each participant, non-parametric K-means clustering was selected to avoid overfitting and restrictive parametric assumptions. Participants were represented in a two-dimensional geometric space based on their log-transformed CMI values at the first and second assessments. K-means clustering partitions the data by iteratively minimising the Euclidean distance between individual coordinates and the cluster centroids. To objectively determine the optimal number of clusters (*k*) and avoid subjective bias, we employed the elbow method to evaluate the total within-cluster sum of squares (WSS) and clinical interpretability. With increasing *k*, the total WSS exhibits a sharp downward trend, after which the curve levels off and a distinct inflection point (elbow) appears. In CHARLS, four optimal subsets were identified (see Figures 2A,B). For class 1, the CMI ranged from 0.234 to 0.306, representing a persistently low CMI levels; for class2, the CMI ranged from 0.612 to 0.438, representing moderate-lower CM levels; for class3, the CMI ranged from 0.482 to 0.936, representing moderate-higher CMI levels; for class4, the CMI ranged from 1.489 to 1.225, representing persistently high CMI levels. The four optimal subsets were similarly identified in ELSA (see Figures 2C,D). For class 1, the CMI ranged from 0.221 to 0.207, representing a persistently low CMI levels; for class2, the CMI ranged from 0.422 to 0.390, representing moderate-lower CM levels; for class3, the CMI ranged from 0.725 to 0.675, representing moderate-higher CMI levels; for class4, the CMI ranged from 1.317 to 1.249, representing persistently high CMI levels. Four optimal subsets were identified in ELSA (see Figures 2E,F). For class 1, the CMI ranged from 0.311 to 0.334, representing a persistently low CMI levels; for class2, the CMI ranged from 0.698 to 0.757, representing moderate-lower CM levels; for class3, the CMI ranged from 1.446 to 1.631, representing moderate-higher CMI levels; for class4, the CMI ranged from 4.534 to 3.689, representing persistently high CMI levels.

Descriptive analysis of baseline characteristics was performed between the four groups. For non-normally distributed continuous variables expressed as median and interquartile range [M (IQR)], the Kruskal–Wallis test was used to compare differences between the four groups. Categorical variables were expressed as frequencies and percentages, and between-group comparisons were performed using the Chi-square test. For eligible participants included in the final analysis cohort, missing values in baseline covariates (the specific missing rates of which are shown in Supplementary Figure S2) were imputed using the non-parametric random forest imputation algorithm from the R package “missForest”. This machine-learning-based interpolation strategy was specifically applied to covariates, as it can effectively handle both continuous and categorical data and automatically model complex non-linear interactions without imposing strict parametric distribution assumptions.

Cox proportional models were employed to calculate Hazard Ratios (HR) and 95% Confidence Intervals (95% CI) to assess the association between cumulative CMI and CMI change patterns and incident CVD risk. Restricted cubic spline (RCS) analysis was used to account for the nonlinear relationship of cumulative CMI. Three models were constructed: model 1 was unadjusted for any covariates, model 2 adjusted for age, sex, BMI, education, marital status, smoking status, and drinking status. Model 3 continued from model 2 by adjusting for SBP, DBP, CRP, total cholesterol, and LDL-C. Subgroup analyses were performed to further explore the interactions between CMI and age (<60 or ≥60 years), sex, BMI, diabetes status, and hypertension (SBP ≥ 140 or DBP ≥ 90 mmHg). Furthermore, to explore whether the observed longitudinal associations were predominantly driven by baseline CMI levels or by temporal changes over time, we performed supplementary comparative Cox regression modeling using the fully adjusted Model 3 covariate set. Specifically, we evaluated and compared two additional regression specifications: Model A, which included static baseline CMI alone; and Model B, which evaluated baseline CMI mutually adjusted for the absolute temporal change (ΔCMI=CMI_2−CMI_1).

All statistical analyses were conducted using R software (version 4.3.2). A two-tailed alpha value of 0.05 considered statistically significant.

## Results

### Characteristics of participants

In this study, 4,450 participants with a median age of 58.0 (52.0–64.0) years, with 54.0% males from the CHARLS cohort, and 3,849 participants with a median age of 60.0 (IQR: 56.0–68.0) years, 57.9% males from the ELSA cohort, and 6,478 patients with a median age of 62.0 (IQR: 57.0–68.0) years, 44.5% males from the EMR datasets. The median follow-up durations for CHARLS, ELSA, and EMR were 5.0 (IQR: 5.0–5.0) years, 8.0 (IQR: 8.0–8.0) years, and 37.3 (IQR: 36.8–38.0) months, respectively. In CHARLS, 861 (41.4 per 1,000-person year) participants eventually incident CVD, including 620 (30.4 per 1,000-person year) participants incident heart disease and 355 (16.7 per 1,000-person year) participants incident stroke. In ELSA, 374 (13.4 per 1,000-person year) participants eventually incident CVD, including 252 (8.9 per 1,000-person year) participants incident heart disease, and 143 (5.0 per 1,000-person year) participants incident stroke. In EMR dataset, 980 (54.4 per 1,000-person year) patients incident CVD, including 651 (35.7 per 1,000-person year) patients incident heart disease, and 412 (22.4 per 1,000-person year) participants incident stroke. The demographic characteristics of the participants are presented in Supplementary Table S1. Compared to class 1, participants in the other class had higher BMI, SBP, DBP, total cholesterol, CRP, HbA1c, LDL-C and cumulative CMI, as well as higher proportions of diabetes and males in the CHARLS dataset. Additionally, the ELSA dataset exhibited higher proportions of older adults (≥60 years), females, individuals with lower education, and smoker.

### Association between CMI change patterns and incident CVD

Table 1 presents the association between CMI change pattern groups and the incident CVD assessed using Cox models. After adjusting for various potential confounders, compared to class 1, the HR for incident CVD with 95% CI were 1.34 (95% CI: 1.09 to 1.64) for class 2, 1.55 (95% CI: 1.26 to 1.91) for class 3, and 1.82 (95% CI: 1.47 to 2.26) for class 4 in CHARLS. However, in the ELSA, only class 4 was statistically significant compared to class 1, with an HR of 1.92 (95% CI: 1.30 to 2.85). In our EMR dataset, Our EMR data similarly showed results consistent with the CHARLS study, with HRs of 1.55 (95% CI: 1.18 to 2.04), 1.72 (95% CI: 1.29 to 2.28), and 2.00 (95% CI: 1.45 to 2.76) for Class 2–4 groups, respectively, compared to the Class 1 group. This implies that higher CMI is associated with incident CVD. In the secondary outcome analysis, similar results were observed for heart disease and stroke, namely that higher CMI trajectory groups were associated with an increased risk of heart disease and stroke (see Supplementary Table S2).

**Table 1:** Association between cardiometabolic index and risk of cardiovascular disease.

Cluster	Model 1		Model 2		Model 3	
	HR (95% CI)	*P* value	HR (95% CI)	*P* value	HR (95% CI)	*P* value
CHARLS (*n* = 4,450)						
Class 1	Ref		Ref		Ref	
Class 2	1.49 (1.23, 1.82)	<0.001	1.43 (1.17, 1.75)	<0.001	1.34 (1.09, 1.64)	<0.001
Class 3	1.78 (1.45, 2.17)	<0.001	1.69 (1.37, 2.08)	<0.001	1.55 (1.26, 1.91)	<0.001
Class 4	2.13 (1.75, 2.60)	<0.001	1.95 (1.57, 2.39)	<0.001	1.82 (1.47, 2.26)	<0.001
ELSA (*n* = 3,849)						
Class 1	Ref		Ref		Ref	
Class 2	1.41 (0.99, 2.02)	0.057	1.26 (0.88, 1.82)	0.212	1.22 (0.84, 1.76)	0.291
Class 3	1.71 (1.21, 2.42)	0.002	1.43 (0.99, 2.07)	0.058	1.32 (0.91, 1.92)	0.140
Class 4	2.51 (1.76, 3.57)	<0.001	2.06 (1.39, 3.04)	<0.001	1.92 (1.30, 2.85)	0.001
EMR (*n* = 6,478)						
Class 1	Ref		Ref		Ref	
Class 2	2.23 (1.71, 2.90)	<0.001	1.99 (1.52, 2.61)	<0.001	1.55 (1.18, 2.04)	0.002
Class 3	3.17 (2.45, 4.10)	<0.001	2.74 (2.09, 3.59)	<0.001	1.72 (1.29, 2.28)	<0.001
Class 4	4.24 (3.20, 5.58)	<0.001	3.72 (2.78, 4.99)	<0.001	2.00 (1.45, 2.76)	<0.001

Definitions of CMI change patterns (Clusters identified by K-means method): Class 1 (Reference): represents persistently low CMI group (baseline to follow-up CMI ranging from 0.234 to 0.306 in CHARLS, 0.221 to 0.207 in ELSA, and 0.311 to 0.334 in EMR); Class 2: represents a moderate-lower CMI group (0.612 to 0.438 in CHARLS, 0.422 to 0.390 in ELSA, and 0.698 to 0.757 in EMR); Class 3: represents a moderate-higher CMI group (0.482 to 0.936 in CHARLS, 0.725 to 0.675 in ELSA, and 1.446 to 1.631 in EMR); Class 4: represents persistently high CMI group (1.489 to 1.225 in CHARLS, 1.317 to 1.249 in ELSA, and 4.534 to 3.689 in EMR).

Adjustment models: Model 1, crude model unadjusted for covariates. Model 2, adjusted for baseline age, sex, BMI, education level, marital status, smoking status, and drinking status. Model 3, further adjusted for baseline diabetes diagnosis, SBP, DBP, CRP, total cholesterol (CHO), and LDL-C.

HR, hazard ratio; CI, confidence interval; Ref, reference group.

**Figure 3:**
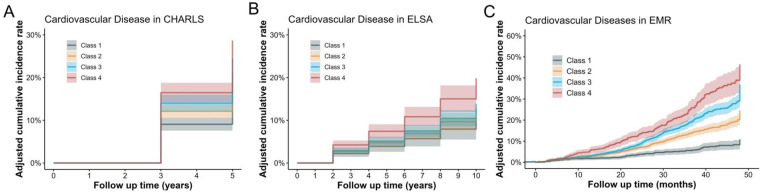
Cumulative incidence curves adjusted for covariates across the three datasets: CHARLS **(A)**, ELSA **(B)**, and EMR **(C)**.

After adjusting for covariates, the cumulative incidence curves similarly revealed that the class 4 group had the highest incidence of CVD across all three datasets, while the class 1 group had the lowest (Figure 3). Similarly, the findings for cumulative incidence were observed for both heart disease and stroke outcomes across the three datasets (see Supplementary Figure S3).

Supplementary analyses evaluating the specific drivers of cardiovascular risk revealed that static baseline evaluations alone were insufficient for long-term risk prediction (Supplementary Table S3). When evaluated in isolation (Model A), baseline CMI was not significantly associated with incident CVD across any of the cohorts after full covariate adjustment (*P* = 0.180 in CHARLS, *P* = 0.651 in ELSA, and *P* = 0.152 in EMR). However, when temporal change (ΔCMI) was incorporated (Model B), longitudinal progression emerged as a significant independent driver of incident CVD in CHARLS (HR = 1.08, 95% CI: 1.01–1.23, *P* = 0.027) and EMR (HR = 1.05, 95% CI: 1.00–1.10, *P* = 0.044), with a strong trend in ELSA (HR = 1.03, 95% CI: 0.99–1.09, *P* = 0.081). Notably, compared to simple linear difference modeling (Model B), our primary K-means cluster classifications (Table 1) provided superior, statistically robust risk stratification across all populations, confirming that composite longitudinal trajectory phenotyping provides essential incremental predictive information beyond static baseline or simple difference metrics.

### Association between cumulative CMI and incident CVD

After adjusting for potential confounders, Cox models showed that in the CHARLS, ELSA, and EMR populations, cumulative CMI was associated with an increased incident CVD risk, with HRs of 1.05 (95% CI: 1.01–1.09), 1.15 (95% CI: 1.07–1.23), and 1.04 (95% CI: 1.01–1.07), respectively (see Supplementary Table S4). Similarly, cumulative CMI was associated with an increased risk of incident heart disease and stroke (*P* < 0.05).

**Figure 4:**
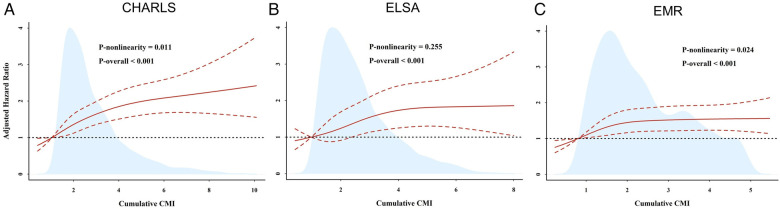
The dose-response relationship between cumulative cardiometabolic index and the risk of incident cardiovascular disease in CHARLS **(A)**, ELSA **(B)**, and EMR **(C)** datasets. The dose-response relationship analysis was conducted using a 4-node restricted cubic spline model, which accounted for the nonlinearity of cumulative CMI, adjusting for all covariates, and using 12.5% as the reference value.

We used a restricted cubic spline (RCS) model with 4 knots to assess the dose-response relationship between cumulative CMI and incident CVD risk. To account for the nonlinearity of cumulative CMI, we designated the 12.5th percentile as the reference value. The RCS results indicated a positive association between increasing cumulative CMI and the increased risk of CVD across all three populations (Figure 4). In the CHARLS and EMR datasets, a nonlinear trend was also observed (*P* values for non-linearity: 0.011 and 0.024), with a sharp initial increase in CVD risk followed by a plateau. Similar findings were observed in the relationship between cumulative CMI and the risk of heart disease and stroke (see Supplementary Figure S4).

### Subgroup analyses

To further evaluate the robustness of the association between CMI change patterns and the risk of incident CVD, we performed comprehensive subgroup analyses stratified by demographic characteristics, lifestyle factors, and baseline comorbidities (see Supplementary Figure S5). In all three independent datasets (CHARLS, ELSA, and EMR), no significant interactions were observed between CMI change groups and any subgroup stratification variables, including sex, age, BMI, education level, marital status, smoking, drinking, diabetes, and hypertension. Specifically, compared with the stable low-risk group (Class 1), individuals in the highest CMI-level group (Class 4) consistently exhibited a higher risk of CVD incidence. Although relatively wide confidence intervals were observed in certain subgroups of the ELSA cohort, this was likely due to the small sample sizes in these strata. However, the trend of increased CVD incidence risk associated with higher CMI classes remained largely consistent across different populations.

## Discussion

We used three independent datasets to investigate the association between long-term changes in CMI levels and CVD in middle-aged and older adults. Our findings indicate that, compared with the persistently low CMI group (Class 1), individuals in higher or persistently elevated CMI groups (class 3 and 4) exhibited a significantly increased risk of incident CVD, heart disease, and stroke across both Chinese and UK populations. Furthermore, this study found that a long-term cumulative CMI elevation was also associated with an increased risk of incident CVD, heart disease, and stroke. The relationship between cumulative CMI and the risk of CVD was nonlinear, characterized by an initial steep rise followed by a plateau.

The CMI is a comprehensive measure determined by obesity and lipid levels. Obesity has been identified as an upstream risk factor for a number of diseases, including diabetes and CVD (28). The physiological mechanisms by which obesity contributes to CVD are numerous and include cytokine release from adipocytes in chronic inflammation, vascular endothelial dysfunction, lipid metabolism disorders, insulin resistance (29). Epidemiologic evidence also confirms the mediating role of atherogenic dyslipidemia, hypertension, and diabetes mellitus in the association between obesity and CVD (30). BMI, the most widely used clinical factor for assessing obesity status, has been extensively studied. However, it still has limitations that tend to give rise to the obesity paradox (31). For two individuals with the same BMI, the shorter person will have a greater WHtR than the taller person. It has been shown that WHtR is preferable to BMI as an indicator of obesity, particularly abdominal obesity, in the prediction of disease, especially cardiovascular disease (32, 33). Therefore, appropriate weight loss and waist circumference reduction may positively impact the reduction of incident CVD.

Insulin resistance (IR) is characterized by reduced sensitivity or responsiveness to the metabolic actions of insulin and plays a significant role in the development of cardiovascular disease (34). The triglyceride to high-density lipoprotein cholesterol (TG/HDL-C) ratio has been validated as a surrogate for IR (35). High TG levels increase the production of very low-density lipoprotein (VLDL) particles, which break down in the bloodstream releasing more free fatty acids and cholesterol, thus impairing the function of reverse HDL-C transport. An elevated TG/HDL-C ratio has been suggested to be associated with a reduction in LDL-C particle size. A study of 40,335 participants from UK biobank revealed that an elevated baseline TG/HDL-C ratio was associated with an increased risk of CVD and coronary heart disease, but not stroke, in a European population (36). A study using the Multiparameter Intelligent Monitoring in Intensive Care (MIMIC) dataset found that the TG/HDL-C ratio increased the 5-year mortality in patients with chronic heart failure and showed good predictive value (37). Another 8-year prospective study of 796 residents found that an increased TG/HDL-C ratio predicted the risk of CVD (38). A prospective study of 9,368 Chinese populations found that higher TG/HDL-C ratios were associated with an increased risk of atherosclerotic cardiovascular disease and stroke after 20 years of follow-up (39). These studies have highlighted the importance of the TG/HDL-C ratio in the prediction of cardiovascular disease.

The CMI, a novel index combining obesity and lipid index, has shown considerable potential for discriminating diabetes. Compared to another marker of lipid accumulation, the lipid accumulation product (LAP), the combination of TG and waist circumference better discriminates hyperglycemia or diabetes in men (11). A cross-sectional survey study of 9,008 participants from the United States found that the risk of congestive heart failure increased significantly with rising CMI levels, demonstrating good predictive value (18). A study using the NHANES dataset by Zhu et al. found that CMI performed better than BMI and WHtR in predicting heart failure (40). In addition, another study involving 11,345 rural Chinese residents in a cross-sectional study found that each SD increase in CMI increased the probability of ischemic stroke by 14% and 18% in males and females, respectively (41). Li et al. also explored the relationship between the combined effects of CMI and inflammation on the risk of new onset strokes, and found a significant association (19). However, most of these studies did not employ cohort studies, and none considered the effect of long-term changes in CMI levels on CVD. Our findings demonstrate that long-term higher CMI levels are independently associated with an increased risk of incident CVD.

Notably, our multivariate analysis revealed that, although persistently high CMI levels (class 4) were significant predictors of incident CVD across all study populations, in the UK ELSA cohort, moderate CMI levels (class 2 and 3) were not statistically significantly associated with CVD, whereas a dose-dependent trend of increased risk was observed in the two Chinese cohorts (CHARLS and EMR). Several potential biological mechanisms may account for these observed differences. There are significant differences between populations in terms of susceptibility to visceral obesity and insulin resistance. Epidemiological and physiological studies consistently indicate that, compared with European populations, East Asian populations are more prone to visceral fat accumulation, ectopic lipid deposition and metabolic dysfunction at lower absolute waist circumference and BMI thresholds (42, 43). Consequently, the metabolic disturbances reflected by the CMI may lead to more rapid vascular endothelial damage and atherosclerosis in Chinese adults. Secondly, differences in access to healthcare are likely to influence the long-term progression of the disease. The ELSA cohort represents a UK population with broad access to primary healthcare interventions, including the early initiation of cardioprotective medications, particularly statins and antihypertensive drugs. Statins actively suppress triglyceride levels, modulate pro-atherosclerotic dyslipidaemia and stabilise vascular inflammation, thereby effectively slowing the progression from moderately elevated CMI (class 2 and 3) to CVD events (44). Nevertheless, the independent and significant risk associated with the class 4 CMI group across all three cohorts confirms that persistently elevated CMI levels are associated with CVD incidence, regardless of population background.

The concept of cumulative levels has been widely discussed and utilized in other studies that account for longitudinal changes over time (45, 46). However, no significant association between CMI and incident stroke was found in the UK population. This may be related to ethnic differences, but further validation is required. Our study also found that, in the Chinese population, CMI in class 2 was initially higher than in class 3 but decreased below class 3 after long-term follow-up, at which time a trend toward a lower risk of CVD was found. Similarly, in the UK population, class 3 was associated with a slightly lower CMI and a reduced risk of CVD. In class 4, a decrease in CVD risk was found at a cumulative CMI < 5.5, followed by an increase. These findings imply that a controlled downward trend in CMI is likely to reduce the risk of CVD. However, additional evidence is required to support this hypothesis.

A pivotal insight generated by our supplementary modeling is that long-term cardiovascular risk is driven predominantly by dynamic changes over time rather than static baseline levels. Remarkably, after controlling for conventional cardiovascular risk factors, baseline CMI failed to independently predict incident CVD across all three cohorts. When temporal change (ΔCMI) and K-means trajectory classifications were introduced, predictive capability and statistical significance were restored. Moreover, while simple linear change (ΔCMI) assumes uniform additive risks regardless of starting burden, our K-means clustering successfully unified initial biological load with longitudinal directionality. This explains why trajectory clustering achieved superior risk stratification reaffirming that continuous particularly in the ELSA cohort, long-term monitoring of CMI trajectories is clinically indispensable for primary cardiovascular prevention.

Our research has several advantages. First, our study considered the association between long-term CMI change patterns and cumulative CMI levels with the risk of incident CVD in middle-aged and older adult. CMI levels measured at long-term follow-up were clustered into four groups representing different CMI control levels. Second, we obtained data from two national prospective surveys across different countries and analyzed them in observational studies, adjusting for multiple confounders. The findings are reliable and generalizable. Finally, this study accounted for the nonlinear relationship of the incidence of CVD. As the parameters required to calculate the CMI can all be obtained through routine clinical examinations, the translation of continuous CMI monitoring into operational clinical practice is of far-reaching significance. The K-means clustering ranges and dose-response curves derived from our data provide scientific guidance for clinical monitoring. Nevertheless, as absolute diagnostic thresholds may be influenced by population heterogeneity, age stratification and racial differences, dedicated diagnostic accuracy trials and clinical guidelines will be required in the future to establish universally standardised thresholds for CMI stratification.

This study also has several limitations. Firstly, when synthesising findings from different data sources, one must acknowledge the significant heterogeneity of the populations and the inherent selection bias. Specifically, whilst CHARLS and ELSA are prospective cohorts of community residents, the electronic EMR cohort comprises patients seeking outpatient or inpatient care. Patients seeking medical care naturally exhibit a stronger willingness to seek treatment and have a higher prevalence of underlying clinical comorbidities; this explains why the higher rates of CMI and CVD incidence observed in the EMR dataset were significantly higher than those in the community cohort. Although we performed rigorous multivariable adjustments to control for potential confounding factors, we cannot completely rule out unmeasured clinic-based selection bias. Nevertheless, we observed consistent positive dose–response findings in both the low-risk community screening setting and the high-risk hospital setting, highlighting the robust and universal clinical applicability of CMI across various healthcare settings. Secondly, the stringent population screening criteria resulted in reduced sample sizes in each cohort, which may have introduced a degree of sampling error. Thirdly, differences in the definition of outcomes may introduce bias. Community-based cohort studies (CHARLS and ELSA) rely on regular self-reports by respondents regarding medical diagnoses, obtained through structured home interviews, which may be subject to recall bias. EMR datasets utilise clinical diagnosis codes; whilst these offer greater specificity, they may omit asymptomatic events or those diagnosed outside the reporting hospital network. Finally, our longitudinal analyses and K-means clusters were constructed using only two CMI measurements per participant across all cohorts. Although assessing two distinct time points can successfully capture directional changes and time-weighted cumulative exposure, it cannot fully describe complex long-term trajectories. Therefore, the findings of this study should be understood as reflecting transitions and cumulative exposure between two points, rather than continuous dynamic trajectories. Future prospective studies are needed, incorporating multiple consecutive assessments and utilising trajectory modelling or joint modelling, to comprehensively characterise the continuous dynamic evolution of CMI and optimise the prediction of cardiovascular risk.

## Conclusion

This study utilized three prospective datasets from two countries, revealing that middle-aged and older adults with persistently high or uncontrolled CMI levels were more likely to develop CVD compared to those with persistently low CMI levels. Higher cumulative CMI was also associated with an increased risk of incident CVD. Therefore, CMI should be used as a simple indicator for the early identification of CVD in clinical practice. Our study supports the long-term monitoring CMI levels, particularly in older adults, to promote the health management and primary prevention of CVD.

## Data Availability

Publicly available datasets were analyzed in this study. This data can be found here: Publicly available datasets were analyzed in this study. This data can be found here: CHARLS (https://charls.pku.edu.cn/en/); ELSA (https://www.elsa-project.ac.uk/). The data will be made available on request.
