# Supplementary material for: Association between cardiometabolic index change patterns and incident cardiovascular disease in middle-aged and older adults
Source: Front Cardiovasc Med. 2026 Jul 31;13:1845385. doi: 10.3389/fcvm.2026.1845385 (PMC13472845; doi:10.3389/fcvm.2026.1845385)
Supplement: Supplementary file 1 [file Datasheet1.docx]

**Association Between Cardiometabolic Index Change Patterns and Incident Cardiovascular Disease in Middle-Aged and Older Adults**

**Legends:**

**Table S1** Baseline characteristics of four class participants.

**Table S2** Association between cardiometabolic index and risk of cardiovascular disease, heart disease and stroke.

**Table S3** Association between baseline CMI, CMI change (∆CMI), and the risk of incident cardiovascular disease.

**Table S4** Association between cumulative CMI and incident CVD, heart disease and stroke risk.

**Figure S1** The design procedure for this study. There was a total of two biomarker tests in CHARLS and three in ELSA. Individuals who received their first biomarker test at wave 2 and wave 4 were included in ELSA.

**Figure S2** The proportion of missing value in CHARLS, ELSA, and EMR datasets.

**Figure S3** Cumulative incidence curves for heart disease (A, C) and stroke (B, D) based on model 3 (full covariate adjustment) in both the CHARLS (A, B), ELSA (C, D), and EMR (E, F) datasets.

**Figure S4** Association between cumulative cardiometabolic index and risk of heart disease (A, B, C) and stroke (D, E, F). Nonlinear relationships were considered using restricted cubic spline based on Cox proportional risk modeling in CHARLS, ELSA, and EMR datasets.

**Figure S5** Association between cardiometabolic index and risk of cardiovascular disease in each subgroup population, using Cox model adjusted for all covariates in CHARLS, ELSA, and EMR datasets.

**Table S1** Baseline characteristics of four class participants.

| Characteristics | CHARLS (N = 4450) | | | | |  | ELSA (N = 3849) | | | | |  | EMR (N = 6478) | | | | |
| --- | --- | --- | --- | --- | --- | --- | --- | --- | --- | --- | --- | --- | --- | --- | --- | --- | --- |
|  | Class 1 | Class 2 | Class 3 | Class 4 | P value |  | Class 1 | Class 2 | Class 3 | Class 4 | P value |  | Class 1 | Class 2 | Class 3 | Class 4 | P value |
|  | N = 1292 | N = 1258 | N = 968 | N = 932 |  |  | N = 719 | N = 1188 | N = 1203 | N = 739 |  |  | N = 1077 | N = 2340 | N = 2186 | N = 875 |  |
| Age, n(%) |  |  |  |  | 0.001 |  |  |  |  |  | 0.01 |  |  |  |  |  | <0.001 |
| < 60 | 706 (54.64) | 711 (56.52) | 595 (61.47) | 573 (61.48) |  |  | 371 (51.60) | 544 (45.79) | 530 (44.06) | 356 (48.17) |  |  | 316 (29.34) | 803 (34.32) | 943 (43.14) | 419 (47.89) |  |
| ≥ 60 | 586 (45.36) | 547 (43.48) | 373 (38.53) | 359 (38.52) |  |  | 348 (48.40) | 644 (54.21) | 673 (55.94) | 383 (51.83) |  |  | 761 (70.66) | 1537 (65.68) | 1243 (56.86) | 456 (52.11) |  |
| Gender, n(%) |  |  |  |  | <0.001 |  |  |  |  |  | <0.001 |  |  |  |  |  | <0.001 |
| Male | 581 (44.97) | 677 (53.82) | 570 (58.88) | 575 (61.70) |  |  | 539 (74.97) | 735 (61.87) | 637 (52.95) | 316 (42.76) |  |  | 651 (60.45) | 1210 (51.71) | 1213 (55.49) | 519 (59.31) |  |
| Female | 711 (55.03) | 581 (46.18) | 398 (41.12) | 357 (38.30) |  |  | 180 (25.03) | 453 (38.13) | 566 (47.05) | 423 (57.24) |  |  | 426 (39.55) | 1130 (48.29) | 973 (44.51) | 356 (40.69) |  |
| BMI, n(%) |  |  |  |  | <0.001 |  |  |  |  |  | <0.001 |  |  |  |  |  | <0.001 |
| < 25 Kg/m^2^ | 1174 (90.87) | 927 (73.69) | 623 (64.36) | 413 (44.31) |  |  | 446 (62.03) | 423 (35.61) | 235 (19.53) | 64 (8.66) |  |  | 644 (59.80) | 1451 (62.01) | 1220 (55.81) | 358 (40.91) |  |
| 25 - 30 Kg/m^2^ | 107 (8.28) | 290 (23.05) | 293 (30.27) | 438 (47.00) |  |  | 238 (33.10) | 566 (47.64) | 596 (49.54) | 335 (45.33) |  |  | 353 (32.78) | 614 (26.24) | 715 (32.71) | 417 (47.66) |  |
| ≥ 30 Kg/m^2^ | 11 (0.85) | 41 (3.26) | 52 (5.37) | 81 (8.69) |  |  | 35 (4.87) | 199 (16.75) | 372 (30.92) | 340 (46.01) |  |  | 80 (7.43) | 275 (11.75) | 251 (11.48) | 100 (11.43) |  |
| Education, n(%) |  |  |  |  | 0.13 |  |  |  |  |  | <0.001 |  |  |  |  |  | <0.001 |
| Primary or lower | 924 (71.52) | 895 (71.14) | 651 (67.25) | 656 (70.39) |  |  | 376 (52.29) | 671 (56.48) | 743 (61.76) | 471 (63.73) |  |  | 633 (58.77) | 1607 (68.68) | 1498 (68.53) | 601 (68.69) |  |
| Secondary or higher | 368 (28.48) | 363 (28.86) | 317 (32.75) | 276 (29.61) |  |  | 343 (47.71) | 517 (43.52) | 460 (38.24) | 268 (36.27) |  |  | 444 (41.23) | 733 (31.32) | 688 (31.47) | 274 (31.31) |  |
| Marital status, n(%) |  |  |  |  | 0.774 |  |  |  |  |  | 0.666 |  |  |  |  |  | <0.001 |
| Spousal | 126 (9.75) | 122 (9.70) | 94 (9.71) | 80 (8.58) |  |  | 179 (24.90) | 316 (26.60) | 297 (24.69) | 195 (26.39) |  |  | 254 (23.58) | 349 (14.91) | 254 (11.62) | 75 (8.57) |  |
| Others | 1166 (90.25) | 1136 (90.30) | 874 (90.29) | 852 (91.42) |  |  | 540 (75.10) | 872 (73.40) | 906 (75.31) | 544 (73.61) |  |  | 823 (76.42) | 1991 (85.09) | 1932 (88.38) | 800 (91.43) |  |
| Smoking, n(%) |  |  |  |  | <0.001 |  |  |  |  |  | <0.001 |  |  |  |  |  | <0.001 |
| No | 711 (55.03) | 800 (63.59) | 630 (65.08) | 613 (65.77) |  |  | 347 (48.26) | 536 (45.12) | 510 (42.39) | 256 (34.64) |  |  | 491 (45.59) | 1206 (51.54) | 1307 (59.79) | 565 (64.57) |  |
| Yes | 581 (44.97) | 458 (36.41) | 338 (34.92) | 319 (34.23) |  |  | 372 (51.74) | 652 (54.88) | 693 (57.61) | 483 (65.36) |  |  | 586 (54.41) | 1134 (48.46) | 879 (40.21) | 310 (35.43) |  |
| Current drinking, n(%) |  |  |  |  | <0.001 |  |  |  |  |  | 0.001 |  |  |  |  |  | <0.001 |
| No | 750 (58.05) | 846 (67.25) | 670 (69.21) | 670 (71.89) |  |  | 33 (4.59) | 62 (5.22) | 76 (6.32) | 67 (9.07) |  |  | 150 (13.93) | 948 (40.51) | 1282 (58.65) | 582 (66.51) |  |
| Yes | 542 (41.95) | 412 (32.75) | 298 (30.79) | 262 (28.11) |  |  | 686 (95.41) | 1126 (94.78) | 1127 (93.68) | 672 (90.93) |  |  | 927 (86.07) | 1392 (59.49) | 904 (41.35) | 293 (33.49) |  |
| Diabetes, n(%) |  |  |  |  | <0.001 |  |  |  |  |  | <0.001 |  |  |  |  |  | <0.001 |
| No | 1253 (96.98) | 1205 (95.79) | 934 (96.49) | 866 (92.92) |  |  | 709 (98.61) | 1161 (97.73) | 1154 (95.93) | 694 (93.91) |  |  | 1060 (98.42) | 2277 (97.31) | 2082 (95.24) | 814 (93.03) |  |
| Yes | 39 (3.02) | 53 (4.21) | 34 (3.51) | 66 (7.08) |  |  | 10 (1.39) | 27 (2.27) | 49 (4.07) | 45 (6.09) |  |  | 17 (1.58) | 63 (2.69) | 104 (4.76) | 61 (6.97) |  |
| SBP, mmHg | 121 (110, 134) | 128 (114, 141) | 126 (114, 139) | 130 (119, 146) | <0.001 |  | 126 (115, 139) | 130 (120, 141) | 133 (123, 145) | 134 (125, 145) | <0.001 |  | 127.00 (115.50, 139.00) | 126.33 (114.67, 140.00) | 129.00 (116.00, 141.92) | 131.00 (119.33, 146.42) | <0.001 |
| DBP, mmHg | 72 (64, 79) | 74 (67, 83) | 75 (69, 83) | 77 (70, 86) | <0.001 |  | 73 (68, 81) | 75 (69, 82) | 77 (70, 84) | 78 (71, 84) | <0.001 |  | 73.50 (66.50, 80.50) | 73.68 (66.67, 81.33) | 75.67 (68.67, 83.33) | 78.00 (70.33, 86.67) | <0.001 |
| Total cholesterol, mmol/L | 3.24 (2.89, 3.63) | 3.26 (2.85, 3.70) | 3.36 (2.95, 3.78) | 3.48 (3.07, 3.93) | <0.001 |  | 6.00 (5.30, 6.60) | 6.00 (5.20, 6.70) | 6.00 (5.20, 6.80) | 6.00 (5.30, 6.90) | 0.087 |  | 0.08 (0.06, 0.10) | 2.72 (0.08, 3.33) | 3.16 (2.56, 3.64) | 3.42 (2.96, 3.89) | <0.001 |
| CRP, mg/L | 0.72 (0.43, 1.50) | 0.93 (0.52, 1.90) | 0.99 (0.56, 2.01) | 1.32 (0.73, 2.53) | <0.001 |  | 1.00 (0.50, 2.00) | 1.50 (0.70, 2.80) | 2.10 (1.10, 4.30) | 2.60 (1.40, 4.95) | <0.001 |  | 1.00 (0.50, 2.20) | 1.10 (0.54, 2.47) | 1.16 (0.62, 2.50) | 1.39 (0.74, 2.70) | <0.001 |
| LDL-C, mmol/L | 2.81 (2.36, 3.31) | 2.95 (2.43, 3.60) | 3.16 (2.61, 3.75) | 2.98 (2.40, 3.68) | <0.001 |  | 3.50 (3.00, 4.05) | 3.70 (3.10, 4.30) | 3.80 (3.00, 4.40) | 3.60 (2.90, 4.29) | 0.001 |  | 0.05 (0.03, 0.08) | 2.21 (0.06, 2.99) | 2.84 (2.05, 3.54) | 2.76 (2.02, 3.43) | <0.001 |
| CMI 1 | 0.23 (0.18, 0.29) | 0.55 (0.44, 0.73) | 0.47 (0.35, 0.61) | 1.29 (1.00, 1.84) | <0.001 |  | 0.22 (0.17, 0.26) | 0.40 (0.33, 0.49) | 0.68 (0.57, 0.85) | 1.25 (0.98, 1.60) | <0.001 |  | 0.30 (0.23, 0.38) | 0.65 (0.51, 0.84) | 1.35 (1.03, 1.76) | 3.54 (2.60, 5.25) | <0.001 |
| CMI 2 | 0.29 (0.22, 0.37) | 0.44 (0.35, 0.53) | 0.84 (0.71, 1.07) | 1.11 (0.82, 1.51) | <0.001 |  | 0.20 (0.16, 0.25) | 0.37 (0.30, 0.46) | 0.64 (0.52, 0.80) | 1.15 (0.95, 1.52) | <0.001 |  | 0.32 (0.24, 0.41) | 0.71 (0.56, 0.89) | 1.46 (1.13, 1.95) | 3.10 (2.30, 4.53) | <0.001 |
| Cumulative CMI | 1.10 (0.88, 1.27) | 1.98 (1.69, 2.44) | 2.75 (2.22, 3.27) | 5.03 (4.08, 6.37) | <0.001 |  | 0.88 (0.71, 1.02) | 1.61 (1.38, 1.85) | 2.74 (2.40, 3.13) | 4.86 (4.23, 5.85) | <0.001 |  | 1.32 (1.00, 1.61) | 2.84 (2.36, 3.42) | 5.79 (4.78, 7.23) | 9.14 (7.63, 10.11) | <0.001 |
| CVD, n(%) |  |  |  |  |  |  |  |  |  |  |  |  |  |  |  |  | <0.001 |
| No | 1123 (86.92) | 1022 (81.24) | 754 (77.89) | 690 (74.03) | <0.001 |  | 676 (94.02) | 1088 (91.58) | 1080 (89.78) | 631 (85.39) | <0.001 |  | 1009 (93.69) | 2041 (87.22) | 1776 (81.24) | 672 (76.80) |  |
| Yes | 169 (13.08) | 236 (18.76) | 214 (22.11) | 242 (25.97) |  |  | 43 (5.98) | 100 (8.42) | 123 (10.22) | 108 (14.61) |  |  | 68 (6.31) | 299 (12.78) | 410 (18.76) | 203 (23.20) |  |
| Heart disease, n(%) |  |  |  |  |  |  |  |  |  |  |  |  |  |  |  |  | <0.001 |
| No | 1148 (90.75) | 1041 (85.75) | 782 (84.36) | 711 (79.35) | <0.001 |  | 693 (96.38) | 1124 (94.61) | 1123 (93.35) | 657 (88.90) | <0.001 |  | 1034 (96.01) | 2137 (91.40) | 1900 (87.32) | 741 (84.98) |  |
| Yes | 117 (9.25) | 173 (14.25) | 145 (15.64) | 185 (20.65) |  |  | 26 (3.62) | 64 (5.39) | 80 (6.65) | 82 (11.10) |  |  | 43 (3.99) | 201 (8.60) | 276 (12.68) | 131 (15.02) |  |
| Stroke, n(%) |  |  |  |  |  |  |  |  |  |  |  |  |  |  |  |  | <0.001 |
| No | 1229 (95.35) | 1166 (92.83) | 854 (88.68) | 830 (89.63) | <0.001 |  | 699 (97.22) | 1147 (96.55) | 1155 (96.01) | 705 (95.40) | 0.277 |  | 1051 (97.59) | 2228 (95.21) | 2003 (91.67) | 781 (89.46) |  |
| Yes | 60 (4.65) | 90 (7.17) | 109 (11.32) | 96 (10.37) |  |  | 20 (2.78) | 41 (3.45) | 48 (3.99) | 34 (4.60) |  |  | 26 (2.41) | 112 (4.79) | 182 (8.33) | 92 (10.54) |  |

**Notes:** BMI=body mass index; SBP=systolic blood pressure; DBP=diastolic blood pressure; CRP=C-reactive protein; LDL-C=low-density lipoprotein cholesterol; CMI=cardiometabolic index; CVD=Cerebrovascular disease. Continuous variables were expressed as Median (interquartile range (IQR)) in case of abnormal distribution and compared between three groups by Kruskal-Wallis test. Categorical variables are presented as counts (percentages) and compared by Chi-square test

**Table S2** Association between cardiometabolic index and risk of cardiovascular disease, heart disease and stroke.

| Event | Cluster | Model 1 | | Model 2 | | Model 3 | |
| --- | --- | --- | --- | --- | --- | --- | --- |
|  |  | HR (95% CI) | P value | HR (95% CI) | P value | HR (95% CI) | P value |
| **Heart disease** |  |  |  |  |  |  |  |
| **CHARLS (n = 4302)** | |  |  |  |  |  |  |
|  | Class 1 | Ref |  | Ref |  | Ref |  |
|  | Class 2 | 1.62 (1.27, 2.03) | <0.001 | 1.51 (1.19, 1.92) | 0.001 | 1.43 (1.27, 1.82) | 0.003 |
|  | Class 3 | 1.75 (1.37, 2.23) | <0.001 | 1.62 (1.26, 2.08) | <0.001 | 1.50 (1.17, 1.94) | 0.002 |
|  | Class 4 | 2.37 (1.89, 2.99) | <0.001 | 2.07 (1.61, 2.66) | <0.001 | 2.00 (1.55, 2.58) | <0.001 |
| **ELSA (n = 3849)** | |  |  |  |  |  |  |
|  | Class 1 | Ref |  | Ref |  | Ref |  |
|  | Class 2 | 1.50 (0.95, 2.36) | 0.084 | 1.26 (0.79, 2.01) | 0.326 | 1.22 (0.76, 1.95) | 0.410 |
|  | Class 3 | 1.84 (1.18, 2.87) | 0.007 | 1.39 (0.87, 2.23) | 0.163 | 1.27 (0.79, 2.05) | 0.316 |
|  | Class 4 | 3.14 (2.02, 4.88) | <0.001 | 2.23 (1.37, 3.62) | 0.001 | 2.04 (1.25, 3.32) | 0.004 |
| **EMR (n = 6463)** |  |  |  |  |  |  |  |
|  | Class 1 | Ref |  | Ref |  | Ref |  |
|  | Class 2 | 2.42 (1.74, 3.37) | <0.001 | 2.05 (1.47, 2.87) | <0.001 | 1.63 (1.16, 2.29) | 0.005 |
|  | Class 3 | 3.40 (2.47, 4.69) | <0.001 | 2.61 (1.87, 3.65) | <0.001 | 1.73 (1.22, 2.45) | 0.002 |
|  | Class 4 | 4.15 (2.94, 5.86) | <0.001 | 3.10 (2.16, 4.44) | <0.001 | 1.88 (1.28, 2.76) | 0.001 |
| **Stroke** |  |  |  |  |  |  |  |
| **CHARLS (n = 4434)** | |  |  |  |  |  |  |
|  | Class 1 | Ref |  | Ref |  | Ref |  |
|  | Class 2 | 1.56 (1.13, 2.16) | 0.007 | 1.56 (1.12, 2.17) | 0.009 | 1.45 (1.04, 2.03) | 0.028 |
|  | Class 3 | 2.51 (1.83, 3.44) | <0.001 | 2.54 (1.84, 3.52) | <0.001 | 2.36 (1.70, 3.28) | <0.001 |
|  | Class 4 | 2.31 (1.68, 3.20) | <0.001 | 2.26 (1.60, 3.19) | <0.001 | 2.02 (1.42, 2.86) | <0.001 |
| **ELSA (n = 3849)** | |  |  |  |  |  |  |
|  | Class 1 | Ref |  | Ref |  | Ref |  |
|  | Class 2 | 1.22 (0.72, 2.09) | 0.461 | 1.21 (0.70, 2.09) | 0.500 | 1.13 (0.65, 1.96) | 0.678 |
|  | Class 3 | 1.39 (0.83, 2.34) | 0.215 | 1.39 (0.80, 2.42) | 0.246 | 1.28 (0.73, 2.25) | 0.382 |
|  | Class 4 | 1.61 (0.92, 2.79) | 0.094 | 1.75 (0.95, 3.22) | 0.074 | 1.76 (0.95, 3.25) | 0.071 |
| **EMR (n = 6475)** |  |  |  |  |  |  |  |
|  | Class 1 | Ref |  | Ref |  | Ref |  |
|  | Class 2 | 2.10 (1.37, 3.21) | 0.001 | 2.03 (1.32, 3.14) | 0.001 | 1.59 (1.02, 2.48) | 0.039 |
|  | Class 3 | 3.56 (2.36, 5.37) | <0.001 | 3.62 (2.36, 5.56) | <0.001 | 2.28 (1.45, 3.58) | <0.001 |
|  | Class 4 | 4.73 (3.06, 7.31) | <0.001 | 5.12 (3.23, 8.11) | <0.001 | 2.74 (1.65, 4.55) | <0.001 |

Notes: Model 1, crude model. Model 2, adjusted for age, sex, BMI, education, marital, smoking, and drinking status. Model 3, adjusted for age, sex, BMI, education, marital, smoking, drinking status, diabetes, SBP, DBP, CRP, CHO, and LDL-C.

**Table S3** Association between baseline CMI, CMI change ($\Delta\mathrm{CMI}$), and the risk of incident cardiovascular disease.

| Datasets | Full model | |
| --- | --- | --- |
|  | HR (95% CI) | P value |
| **CHARLS** |  |  |
| Model A: CMI 1 | 1.05 (0.98, 1.16) | 0.180 |
| Model B: CMI 1 | 1.05 (1.00, 1.17) | 0.035 |
| Model B: $\Delta\mathrm{CMI}$ | 1.08 (1.01, 1.23) | 0.027 |
| **ELSA** |  |  |
| Model A: CMI 1 | 1.03 (0.82, 1.10) | 0.651 |
| Model B: CMI 1 | 1.04 (0.98, 1.18) | 0.155 |
| Model B: $\Delta\mathrm{CMI}$ | 1.03 (0.99, 1.09) | 0.081 |
| **EMR** |  |  |
| Model A: CMI 1 | 1.03 (0.99, 1.07) | 0.152 |
| Model B: CMI 1 | 1.06 (1.00, 1.10) | 0.026 |
| Model B: $\Delta\mathrm{CMI}$ | 1.05 (1.00, 1.10) | 0.044 |

Notes: All results are based on the full model, adjusted for all covariates, including age, sex, BMI, education, marital, smoking, drinking status, diabetes, SBP, DBP, CRP, CHO, and LDL-C. Model A includes baseline CMI; Model B includes baseline CMI and $\Delta\mathrm{CMI}$.

**Table S4** Association between cumulative CMI and incident CVD, heart disease and stroke risk.

| Event | Model 1 | | Model 2 | | Model 3 | |
| --- | --- | --- | --- | --- | --- | --- |
|  | HR (95% CI) | P value | HR (95% CI) | P value | HR (95% CI) | P value |
| **Cardiovascular disease** |  |  |  |  |  |  |
| CHARLS | 1.08 (1.05, 1.12) | <0.001 | 1.05 (1.01, 1.10) | 0.007 | 1.05 (1.01, 1.09) | 0.027 |
| ELSA | 1.17 (1.10, 1.24) | <0.001 | 1.14 (1.07, 1.23) | <0.001 | 1.15 (1.07, 1.23) | <0.001 |
| EMR | 1.13 (1.10, 1.15) | <0.001 | 1.11 (1.09, 1.14) | <0.001 | 1.04 (1.01, 1.07) | 0.005 |
| **Heart disease** |  |  |  |  |  |  |
| CHARLS | 1.07 (1.02, 1.11) | 0.002 | 1.06 (1.02, 1.11) | 0.005 | 1.05 (1.01, 1.10) | 0.024 |
| ELSA | 1.22 (1.14, 1.30) | <0.001 | 1.19 (1.11, 1.28) | <0.001 | 1.15 (1.07, 1.24) | <0.001 |
| EMR | 1.12 (1.10, 1.15) | <0.001 | 1.09 (1.06, 1.12) | <0.001 | 1.08 (1.05, 1.12) | <0.001 |
| **Stroke** |  |  |  |  |  |  |
| CHARLS | 1.13 (1.08, 1.19) | <0.001 | 1.11 (1.05, 1.18) | <0.001 | 1.11 (1.05, 1.18) | 0.001 |
| ELSA | 1.07 (0.97, 1.18) | 0.181 | 1.12 (0.99, 1.26) | 0.069 | 1.15 (1.01, 1.30) | 0.029 |
| EMR | 1.14 (1.11, 1.18) | <0.001 | 1.15 (1.11, 1.19) | <0.001 | 1.07 (1.03, 1.12) | 0.001 |

Notes: Model 1, crude model. Model 2, adjusted for age, sex, BMI, education, marital, smoking, and drinking status. Model 3, adjusted for age, sex, BMI, education, marital, smoking, drinking status, diabetes, SBP, DBP, CRP, CHO, and LDL-C.

**Figure S1** The design procedure for this study. There was a total of two biomarker tests in CHARLS and three in ELSA. Individuals who received their first biomarker test at wave 2 and wave 4 were included in ELSA.

**Figure S2** The proportion of missing value in CHARLS, ELSA, and EMR datasets.

**Figure S3** Cumulative incidence curves for heart disease (A, C) and stroke (B, D) based on model 3 (full covariate adjustment) in both the CHARLS (A, B), ELSA (C, D), and EMR (E, F) datasets.

**Figure S4** Association between cumulative cardiometabolic index and risk of heart disease (A, B, C) and stroke (D, E, F). Nonlinear relationships were considered using restricted cubic spline based on Cox proportional risk modeling in CHARLS, ELSA, and EMR datasets.

**Figure S5** Association between cardiometabolic index and risk of cardiovascular disease in each subgroup population, using Cox model adjusted for all covariates in CHARLS, ELSA, and EMR datasets.
